# How breaks in nature can affect the users’ wellbeing: an experience based survey during the lockdown: Marco Gola

**DOI:** 10.1093/eurpub/ckac129.708

**Published:** 2022-10-25

**Authors:** M Gola, M Botta, AL D’Aniello, S Capolongo

**Affiliations:** 1 Design & Health Lab, DABC - Politecnico di Milano, Milan, Italy; 2 Monica Botta Architects, Novara, Italy; 3 HUB 4 MIND, Varese, Italy

## Abstract

**Background:**

In the occasion of COVID-19 pandemic in Italy, the life of citizens was greatly disrupted - from healthcare professionals to the smart workers - and consequently also the state of mood. On the basis of the scientific evidences in relation to the relationship between the built environment and health, a research group has promoted an investigation on the benefits that greenery can have on the psychophysical state of the users, especially healthcare staff and users at home.

**Objectives:**

The methodology adopted is the Profile of Mood States, which provides experiential activity in nature - without any technological distraction- to evaluate the benefits on mood. The methodology adopted refer to the shorter version (34 items) designed by prof. Grove at the University of Western Australia. In relation to the COVID-19 pandemic, the experience based questionnaire is differentiated for healthcare staff and general users. The questionnaire is composed of a few questions, to be completed before and after an experience in nature of 20/30 minutes. The investigation requires to be carried out in private gardens, balcony and/or terrace with greenery, public green areas, etc.

**Results:**

300 participants (subdivided into 225 general users and 75 healthcare professionals) took part in the investigations. Data analysis highlighted the higher performances in anxiety, depression, anger, force, fatigue and confusion, in particular for users who had the experience in garden (-50/70%), and among the healthcare staff the best outcomes are related to who did the investigation during or after the workshift (-60/-90%).

**Conclusions:**

Although it is well-known the benefits that nature affects positively on well-being and stress level of users, the investigation underlines that brief breaks in the nature - especially in period of great stress such as pandemic - can influence the well-being and mental health of users.

